# Context Graphs as an Efficient and User-Friendly Method of Describing and Recognizing a Situation in AAL

**DOI:** 10.3390/s140611110

**Published:** 2014-06-25

**Authors:** Andrei Olaru, Adina Magda Florea

**Affiliations:** Computer Science Department, University Politehnica of Bucharest, 313 Splaiul Independentei, 060042 Bucharest, Romania; E-Mail: adina.florea@cs.pub.ro

**Keywords:** ambient assisted living, ambient intelligence, context awareness, context graphs, context patterns, graph matching, situation recognition

## Abstract

In the field of ambient assisted living, the best results are achieved with systems that are less intrusive and more intelligent, that can easily integrate both formal and informal caregivers and that can easily adapt to the changes in the situation of the elderly or disabled person. This paper presents a graph-based representation for context information and a simple and intuitive method for situation recognition. Both the input and the results are easy to visualize, understand and use. Experiments have been performed on several AAL-specific scenarios.

## 1. Introduction

The field of ambient assisted living (AAL) has the goal of developing hardware and software systems that are able contribute to the care for persons that are elderly or disabled (mentally or physically). The main target is the elderly population, which is continuously growing in number, and, more specifically, aging persons that live alone [1,2]. While the human factor cannot be taken out of the loop, such systems can make a significant contribution by reducing the involvement of the caregiver only to those situations that require explicit action or response. Using such systems, elders can live in the comfort of their home while the loved ones do not need to worry about their whereabouts.

There has been a large volume of very diverse work in the field of AAL, with approaches ranging from more intrusive (devices worn by the assisted person, measuring their vital signs) to the less intrusive (e.g., fall detection by sound recognition). Software has been also ranging from simple isolated applications for specific tasks to complex platforms managing a great number of devices and services.

In this context, this paper addresses the core of the AAL system, which is situation recognition, decision upon action and overall intelligent behavior. The approach is based on the idea that an AAL system should use a smaller number of sensors and achieve intelligence by indirect recognition of potentially relevant situations (emergencies or other times when assistance is/may be needed). Intelligent behavior should not only be instantaneous (detect the situation based on perceptions at the moment it happens), but should also use time-related information (situations that have happened in the past leading to a potential problem in the present).

As an unobtrusive system that gathers partial information from sensors and intelligently aggregates it to recognize the situation (context) and to assist the user, an AAL system is an ambient intelligence (or AmI [3–5]) system directed mainly towards the assistance of a single user and customized for his/her specific situation. Central to both AAL and AmI is the ability to recognize the user's context and act accordingly, without failing to take appropriate action, or intruding, or disrupting the user's activity.

A less addressed issue in AAL projects is the relation between the assisted person and the caregiver [6]. There may be formal caregivers, but there may also be informal caregivers, such as relatives, friends, *etc.* As the state of the assisted person may change and as the adaptation of the platform may be necessary, it is important that the caregivers, both formal and informal, are able to adjust the behavior of the system easily. Moreover, when the system decides upon action, it is important that the caregiver is able to understand the decisions taken by the system. This work proposes an approach that addresses this issue, as both the representation of the recognized situation and of the appropriate action, as well as the reasoning process, share a simple foundation and accessible visualizations.

The purpose of this paper is to propose a representation for context information, as well as a means of recognizing context, based on graphs and graph patterns, aimed at usage in AAL scenarios and applications. Graphs, proposed for the representation of context information and recognized situations, are easy to understand and visualize. Graph matching, proposed as a means of recognizing the situation and making decisions, is a process that is easy to picture, and efficient algorithms have been developed for its realization. Moreover, graph patterns allow for defining complete situations that may be only partially matched, leading to proactive behavior in which the system tries to complete a situation that is almost, but not quite, completely detected.

This work is framed by the AmIciTy initiative of creating a context-centered, agent-based distributed platform for AmI applications [7]. The context matching mechanisms presented here integrate with the software agents in the platform, which use graphs to represent the current situation, recognized situations and transferred information. Context matching, and especially timelines, are based on our previously presented (but more simple) context graphs and patterns and context matching algorithm [8]. In this paper, we will concentrate on the context matching process inside a single agent and the advantages such a mechanism offers for AAL applications.

After presenting some related work in the field in the next section, we discuss situation representation in more depth in Section 3. The formal foundation for context graphs, context patterns, context matching and temporal context is presented in Section 4. Section 5 gives some insights on the implementation of the context-matching platform, and Section 6 presents the experimental scenarios and the results of experiments. The last section draws the conclusions.

## 2. Related Work

This section presents works that are related to the key elements of our approach, namely ambient intelligence platforms, the management and modeling of context information, especially the modeling of context information as graphs, and issues related to graph matching.

### 2.1. AmI Platforms for AAL

The development of software and hardware platforms specifically directed to ambient assisted living is a relatively recent endeavor. Most platforms are based on research in ambient intelligence environments and smart homes, but, this time, customized for use by a single (usually) assisted individual [9–11].

One of the things that is easy to see is that most proposed platforms have a very complex architecture that is meant to bring information from a large number of sensors, which communicate using various protocols. Furthermore, most platforms are centralized, relying on a central server in the home that does all of the processing, relying mostly on rules to decide upon the appropriate action. The environment and the user's context are many times represented as tuples of values from sensors, and sometimes ontologies are used to define situations. What is often times missing, though, is intelligence itself [12]. Usually, action is decided based on large amounts of information (including various smart devices, such as smart cups, showers, floors, *etc.*) that would be normally difficult or intrusive to obtain [13–15]. However, there is a strong lack of support for changing situations, for temporal sequences of situations and for easy understanding of how the system works by a less informed caretaker. While research on multi-protocol, multi-device architectures is extremely useful for the case of the smart home, reasoning and true intelligence is always necessary in AAL. We will look into some individual examples.

Lyons *et al.* [13] present an important corpus of use cases for single-inhabitant smart homes, using case-based reasoning to decide on the severity of the case (if any). While the scenarios are very useful, the authors assume that the system is composed of largely smart appliances (including small ones, such as a blender) able to detect the length of the person's shower, the phases of tea-making and the position of various small objects. While this can technically be done, it would be overly complicated and expensive. The Ambient Home Care System (AHCS) [1] proposes a large number of sensors as well: motion detectors, smart carpets, wrist devices and smart walking aids. As stated further on, we try to obtain intelligent behavior using a smaller number of sensors, leading to less intrusiveness and a lower cost of the system. A relevant study on the perception on wearable devices, namely a smart wrist device, has been done by Holzinger *et al.* [16]. Similarly, Sun *et al.* [6] propose an interesting interface to be used for elders in order to interconnect with each other and their carers, but the system is purely reactive, and there is no activity recognition involved; participants must explicitly declare their needs.

We find that reasoning in many AAL applications is lacking, not exploiting at the fullest the information brought by sensors and through communication. For instance, Tapia *et al.* [11] present several agent-based AAL applications and projects that rely on various sensors and protocols (RFID, WiFi, ZigBee) to assist the person. The architecture and the presented infrastructure are valuable, but the system works only as a gatherer of information. Context-aware reasoning by the agents would greatly improve the performance and the features of the system.

Klein *et al.* [17] present the results of the SOPRANO European project (Service-Oriented Programmable Smart Environments for Older Europeans) featuring the SOPRANO Ambient Middleware (SAM). The project features a complex architecture built around a context manager, reasoning using relations between various elements of context, much like in our work, and ontologies. As ontologies do not perform well in the case of dynamic context, we claim that using context matching would improve the performance of reasoning.

The work of Blasco *et al.* [18] presents a complex platform, encompassing a large number of devices and protocols and an interesting architecture for the core of the system. The Context Manager uses a fixed set of rules that are specific to the appliances in the system. While useful as a platform for controlling and gathering information from devices, there is room for improvement in the reasoning and intelligence of the system.

For further implementations, we refer the reader to more complete surveys [10,15], from which one can see that, where intelligent behavior, using AI methods and mechanisms, is implemented, it is done so in a manner that is not easy to adjust or update by a less-informed carer. We address this by proposing mechanisms that use representations that are easier to understand and to use.

### 2.2. Context Awareness and Context Modeling

In context awareness for pervasive computing, infrastructures for the processing of context information [19,20] contain several layers, going from sensors to the application. There are several challenges in building infrastructures for context awareness [21], related, on the one hand, to managing data formats and protocols, and on the other hand, to splitting responsibilities (processing) between devices. Middleware proposed for context-aware services [22,23] are hierarchical, separating context providers from context consumers. This type of infrastructure is useful when the context information comes from the environment and refers to environmental conditions, such as location, temperature, light or weather, also having a simpler representation [24]. Our approach is directed towards an infrastructure that is decentralized, in which each entity/agent has knowledge about the context of its user, and applications are also able to create context information and insert it into the system, combining the features of context providers and consumers [7].

A large number of AmI platforms rely on software agents [25] to handle distributed configurations. However, in doing so, context awareness generally remains centralized, as agents use context servers or centralized context services to store and to provide context information and to perform context-aware reasoning (detection of situations). Here, we can refer platforms such as iDorm [26], LAICA (Laboratory of Ambient Intelligence for a Friendly City) [27] or AmbieAgents [28]. We propose mechanisms to distribute context-aware reasoning and context information, such that each agent handles the information relevant to its activity.

Modeling of context information uses representations that range from tuples to logical, case-based and ontological representations [15,19,29–31]. The most popular approaches are ontologies for representing situations and rules for reasoning, coupled with propositional or predicate logic to represent current context information.

Rules are used to specify the action to perform when a particular set of conditions are met [32,33]. However, rules are not easy to adapt, and their formalisms cannot be easily used by a less informed carer. Defeasible rules are an interesting formalism to define implications that are not necessarily true, accounting for uncertainty and erroneous context information [34].

Ontologies are used in many projects to define situations and to establish the relations among the elements of the context. Several ontologies have been created specifically for use in context-aware computing (e.g., SOUPA [35]). The main criticism regarding ontologies is a lack of support for temporal relations, the lack of dynamicity and the large space and temporal complexity required for ontological reasoning [30]. Solutions have been proposed for integrating temporal information into ontologies [36], for reducing the footprint on resource-constrained devices [37] and for distributing ontologies across devices [38]; however, ontologies remain more appropriate for centralized approaches, where reasoning is performed by powerful devices. The mechanisms we propose are directed towards system distribution, the local storage of context information and local reasoning.

The work of Turner *et al.* shows how context-mediated behavior (CMB) [39] can be used to adapt behavior to cases that have not been encountered before, but share similarities with existing cases, much like we use patterns for context recognition. Their work extends case-based reasoning and is applied in AUV (Autonomous Underwater Vehicles) control [40]. Our research is somewhat similar in behavior, but we use structures that are easy to represent graphically and to visualize.

Another mechanism that can be used for activity recognition is Event Calculus [41], a logic formalism relying on fluents (variables) that can be changed by the occurrence of events at various times. While using this formalism can give good results for small cases [42], the constraints and the situation recognition algorithm make it impractical for situations with a large number of variables.

As we attempt to use graphs for the representation of context information, the work of Sowa [43] on conceptual graphs is especially interesting with respect to our research, but, in our work, we define a textual and graphical representation that is easier to use, and we focus on graph matching for situation detection.

In the field of time-aware graph-based representations, the body of research is small. Ontologies do not handle temporal relations very well, and more work has been done in time-aware RDF (Resource Description Format) or similar representations. We mention the work of Pollack [2] in terms of tagging graph nodes with information on realization intervals and the works on temporal queries on RDF (e.g., [44]). Our work, however, is based more on graph features, integrated with context matching, and easier to visualize. We do not focus only on the validity of relations, but also on graphs that represent a sequence of events.

We find the work of Storf *et al.* [45] very interesting from the point of view of aggregating events into temporal trends. While our work draws from theirs, we are building upon it by coupling temporal awareness with the graph-based representation of context.

Similarly, Fernández-Llatas *et al.* [46] aggregate the recognition of context into workflows to detect outliers and deviation from the normal situation. However, the system only has a unidirectional perception of context, and context information is limited to location. We propose a representation that encompasses not only other types of perceptions, but also associations between facts and the ability of applications to generate context information themselves.

The research group of Diane Cook working on activity detection proposes a method that bears much similarity to our own, in the sense that textual pattern detection is used to detect behavioral patterns in activity data recorded as text [47,48]. By comparison, this work is directed towards activity recognition (rather than detection) and proposes a representation for context/situation information that is easier to read and handle by the carer of the assisted person.

### 2.3. Graph Matching

Graph matching (finding an isomorphism between two graphs) has been used for a great variety of domains, prominently for image analysis, chemistry, document processing and ontology matching, but less in context recognition [49]. Graph matching is an NP-complete problem. In our specific situation, what is needed is the exact and potentially incomplete matching of labeled, directed graphs. Most matching algorithms [49] are focused on unlabeled graphs, meant especially for image recognition. We have taken inspiration from several popular algorithms, such as the ones created by Bron-Kerbosch [50], Larossa [51] and Cordella [52], and we have found that dedicated matching algorithms can be created that, for the specific problem of context matching, offer very satisfactory performance [8].

Kirsch-Pinheiro *et al.* [53] use graph matching for context-aware service selection in order to handle dynamic context; however, they actually match taxonomical graphs and not graphs representing the current situation.

Chein *et al.* [54] use graph matching for visual reasoning (not necessarily context-awareness) using generalization and specialization operations to find if a graph can be transformed into the other. Their research is very useful from the point of view of graph transformation; however, finding matches based on trying various sequences of operations seems far from an efficient method for matching.

## 3. A Perspective on Representing Situation in AAL

The main concern in AAL is for the system to perceive and understand the situation of the assisted person (the ‘user’) and to be able to take appropriate action, which can be one of: offering a suggestion to the user of what s/he could do now, if the user appears to be vague or lost; notifying the/a carer when potentially worrying situations arise, e.g., the assisted person goes to the bathroom too often, or is in the bathroom for too long, or moves erratically around the house; notifying all carers and emergency services when an emergency is detected with certainty.

In this context, it is important to understand what *situation* means, how it can be defined and how a response may be generated. Of equal importance is that the behavior of the system must be customizable by the carer of the person in an easy and accessible way, tuning the system for the particular cases and needs of the assisted person. Moreover, the carer(s) must be able to have a general understanding of the principles behind the reasoning of the system, so as to understand why an action was decided upon by the system and how the system may be tuned in order to fix undesired behaviors.

### 3.1. Physical Environment and Sensors

This research integrates with our AmIciTy initiative [55] to build a context-aware, agent-based platform for AmI and AAL applications. The platform is in the course of being deployed in the Ambient Intelligence Laboratory in our faculty [56,57], funded by the ERRIC FP7 European Project [58]. The laboratory contains various sensors, among which, the most interesting to this research are Microsoft Kinect^TM^ RGB-D cameras, capable of tracking a user's skeletal approximation and position relative to the camera. If only the depth information from the sensor is used, we consider that the privacy of the subject is respected.

The choice of sensor equipment has been guided by our perspective on AAL. Wearable sensors are felt by the person as an intrusion to their privacy, and it may be that the person will dissent, will refuse or will avoid wearing them. Moreover, in a social environment, wearable devices (such as sensors or tracking bracelets) may attract stigmatization, as they show that the person is indeed in need of assistance and cannot fully care for themselves [14,45]. Devices that require action from the assisted person, on the other hand (such as panic buttons), may not be activated in time or at all when there is an emergency. In our view, the solution lies in using only a few, less intrusive sensors that the person can easily adapt to, such as depth cameras and microphones, used to detect the location and posture of the person, and, occasionally, RFID tags, used to detect object use [59]. This way, there is no direct interaction between the person and the sensors, and issues regarding the acceptability of technology by the person are minimized. In the future, when the system will need to interact with the assisted person, usability and acceptance studies will need to be conducted [60].

### 3.2. A Brief Overview of the System Architecture

The AmIciTy platform is an agent-based platform for AmI and AAL applications [7]. It is oriented towards a fully distributed functionality, using few or no centralized components. The platform relies on a context-aware layer that handles locally-stored context information and deals with recognizing situations, integrating interesting information received from other agents and disseminating potentially interesting information to other agents. Each agent resides on a machine close to where its function is needed and maintains only context/situation information that is relevant to its own function. Some agents may receive perceptions from simple devices and do only simple processing; other agents may reside on powerful workstations and handle complex detection tasks, featuring large context graphs and many patterns.

**Figure 1.**
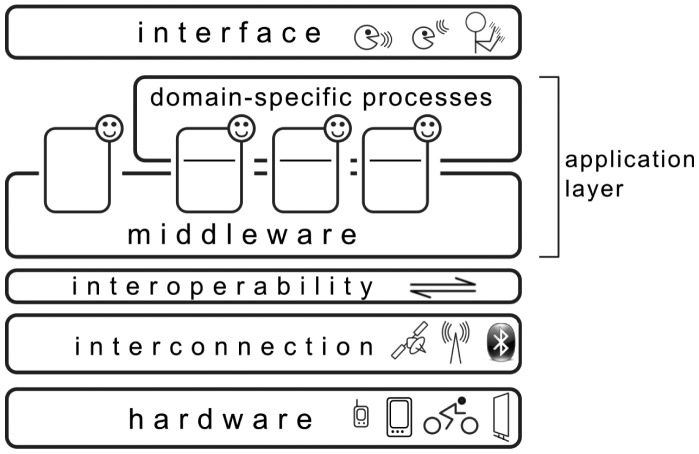
A layered perspective on the AmIciTy platform, featuring agents that are part of the context-aware middleware, but may also contain application-specific behaviors. The architecture has been introduced in previous work [7].

Figure 1 presents a layered perspective on the components of the system (based on the work of Seghrouchni [61]), in which agents reside in a layer dealing with application-specific behavior, but containing a sublayer—the context-awareness middleware—which is generalized so as to be used by any AmI application.

In this paper, we concentrate on describing how context matching can be useful for AAL applications, and the multi-agent system aspect of the platform will not be discussed, as it has been done so in previous work [7]. The presentation will focus on the functionality of only one agent, keeping in mind that the design of the context graphs has been done so that the representation is easy to distribute among multiple agents.

### 3.3. Situation Recognition

We view situation/context as a set of associations between different concepts, a view somewhat inspired by the work of Henriksen and Indulska [62]. The context is formed by the elements that are relevant to the user's activity and can act as the ‘dressing’ of the user's focus [63,64]. Using associations is an improvement over using tuples or properties of context elements and is similar to logical representations, but easier to understand and visualize.

**Figure 2.**
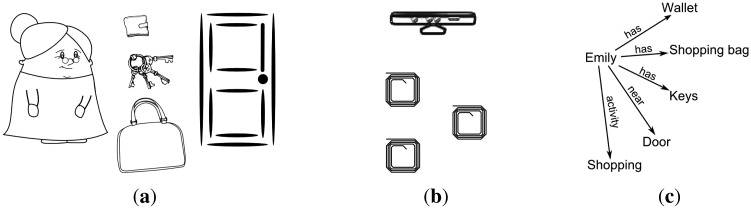
(**a**) Scenario: Emily is near the door, having her wallet, her keys and her shopping bag with her. (**b**) The Kinect sensor detects Emily near the door. The RFID detector near the door detects the tags placed on her wallet, keys and bag. (**c**) The pattern used by the system to detect, based on the given information, that Emily will go shopping. Clip art from http://clker.com under a Creative Commons Zero (CC0) License.

Let us have an example scenario, featuring the fictional character, Emily, who we will use throughout the paper. She is aged 87 and lives alone. We address situations in which Emily is alone in her home. If Emily needs to go out of the house to do some shopping, normally she gets dressed, she takes her wallet, her keys and a shopping bag with her. The situation of “Emily is going out for shopping” is defined by four associations: Emily is dressed in her city clothes; Emily has her wallet with her; Emily has her keys with her; and Emily has a shopping bag with her (see Figure 2). If only some associations are detected (e.g., all, except for the wallet), the user may be in the situation, but something may be wrong (the situation is not complete); Emily has forgotten her wallet. As a result, the system may issue a notification to remind Emily of the missing association.

The above works for instantaneous context, where a situation is detected in the moment when all or most of the associations are present. However, there are cases in which time and sequence play a role. For example, in Emily's home there are RGB-D cameras in every room, except for the bathroom (for privacy concerns), but the system cannot detect Emily anywhere in the house. It may be that Emily is in the bathroom or that Emily has gone out of the house. However, if Emily has been near the bathroom door exactly before disappearing, it is most likely that she went to the bathroom. Another example: if Emily goes to the bathroom and does not come out for one hour, there may be a problem, and the carer must be notified or (if the system has the possibility) the system may try to obtain a response from Emily that she is alright.

**Figure 3.**
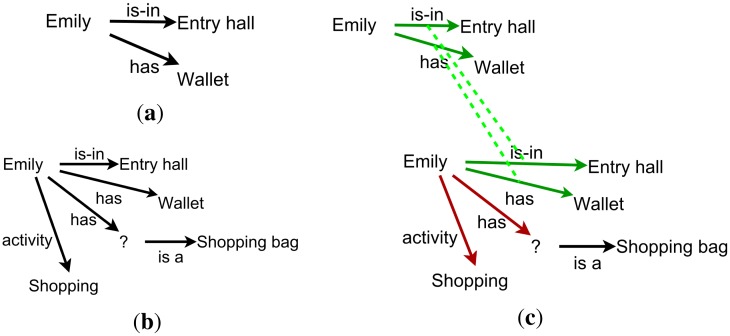
(**a**) A context graph saying Emily is in the hall and has the keys. (**b**) A context pattern describing the situation in which Emily goes shopping, taking her wallet and shopping bag. (**c**) A match between the context graph and context pattern, showing what matches and what is missing.

From the point of view of ease of use, a situation described as associations is easier to define and understand by a carer. Instead of describing rules, the carer just has to describe the situation, which potentially includes actions from the part of the system (e.g., notifications). When the situation is represented as a graph, it becomes even easier. Moreover, if identifying a situation relies on identifying associations between the present context and the desired situation, it is easy for someone to see what exists and what is missing (see, for instance, Figure 3).

**Figure 4.**
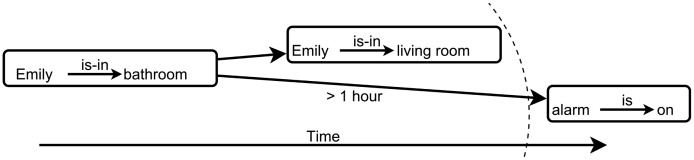
Example of a timeline. The dotted line represents the current moment (“wave”) of time.

For sequences and time-related situations, the easiest for someone is to visualize time as flowing along an axis and various events happening at the right times on this axis. Alternative sequences of events can start from the same starting point, but have different outcomes, depending on what happens along the way. If laid out on an axis, temporal distance (time passed between events) is easy to picture. This is how we have developed the concepts of *timelines*, presented in Section 4.6 (see Figure 4).

From the point of view of artificial intelligence, describing possible complete situations instead of rules can be interpreted as having a goal-driven, cognitive behavior instead of a reactive behavior. Once a situation is sufficiently matched by the current context, the agent can set itself a goal to complete the situation as it is defined, instead of just reacting to perceptions.

Following the reasoning above, we have decided that, especially in the context of an AAL system, used both by the assisted person and by the caregiver(s), the current context would be best represented as a graph of associations/relations between concepts (the context graph). Known situations are represented also as graphs, possibly featuring some generic elements, which can be matched against the context graph to detect the current situation(s). This formal representation is described in depth in Section 4.

## 4. Using Context Graphs and Patters to Represent Situations

In the AAL system, each agent should have a representation of the information that is interesting to it and also the means of detecting what information is interesting to it from the stream of information that it receives. Moreover, it should have a representation of other agents' interests, in order to know whom to inform of potentially interesting information, out of all of the agents that share some context with it.

The aim of the representation defined below is to serve as a simple and flexible way of representing the knowledge about the user's current situation. It must be easy to change, by removing or adding associations, and it must be able to adapt to the storage space: an agent must be able to store some relevant information for its function, even if it has very limited storage capabilities. The representation must also allow the agent to easily aggregate interesting received information with existing information. While the representation has been presented in various forms in previous work, it has never been done as completely and as coherently as in this section.

### 4.1. Current Situation Represented as the Context Graph

In order to represent its beliefs about the current situation, each agent *A* has a *context graph CG_A_* = (*V*, *E*) that contains the information that is currently relevant to its function.

We consider a global set of concepts to be present in the graph:

*Concepts* = *Strings* ∪ *URIs*

In this definition, *Strings* stands for the set of all strings and *URIs* for the set of all valid Universal Resource Identifiers. Nodes must have a non-empty label. There is no taxonomy for the concepts and no way to generalize a concept to another concept (as in the work of Chein *et al.* [54]). However, complex ontologies (or even taxonomies) are not absolutely needed in an environment covering one habitation and the activity of one person. In this case, having a name and a class for each item and activity is enough. For instance, it is sufficient to have $\text{Sleeping}\overset{\text{isa}}{\to }\text{Activity}$ to say what is the class of a node. This can be matched in a pattern? $\overset{\text{isa}}{\to }\text{Activity}$ or, in short, ? *Activity*. If more complexity is needed, taxonomies can be defined in the context graph using multiple $\overset{\text{isa}}{\to }$edges and can be matched using regular expression pattern edges.

We also define a global set of *Relations* between concepts:

*Relations* = *Strings* ∪ *URIs* ∪ {*λ*}

In the relation set, we also have the empty label in order to express unnamed relationships, with no special semantics, except just the association between two concepts.

Using the two sets defined above, we define the context graph as:

*CG_a_* = (*V, E*), where
*V* ⊆ *Concepts* and
*E* = {(*from, to, value, validity*) | *from, to* ∈ *V, value* ∈ *Relations, validity* ∈ (0,1] ∪ {*permanent*}}.

First, it is easy to see that two nodes cannot have the same label. One concept will be present in the graph as only one node. Second, we note that the edges have an additional attribute, which is the *validity* of the edge. We will discuss this in Section 4.5. Multiple edges with the same label may exist in the graph, but not between the same two nodes.

### 4.2. Recognized Situations Represented As Context Patterns

In order to detect relevant information or to find potential problems, an agent has a set of patterns that it matches against its context graph *CG_A_*. These patterns describe situations that are relevant to its activity (we will note with the superscript “*^P^*” the support for generic elements, such as nodes labeled with a question mark):

*Patterns* = {( $G_{s}^{P}$, *persistence*) | s ∈ *PatternNames*, $G_{s}^{P}$*a graph pattern, persistence* ∈ (0,1] ∪ {*permanent*}}

That is, a pattern *s* is defined by a graph pattern $G_{s}^{P}$ and *a persistence* property that gives the validity of edges that are inserted in the context graph as a result of actions (we will talk about actionability a bit later). A graph pattern has the following properties:

$G_{s}^{P}=(V_{s}^{P},E_{s}^{P})$

A graph pattern is just a graph with some generic elements (labeled with a question mark), namely vertices that can match any vertex in the context graph and edges that that can match edges with any label.

$V_{s}^{P}$ = {(*label*, *index*), *label* ∈ *Concepts* ∪ {?}, *index* ≥ 0, *label* = ? ⊕ *index* = 0}, with ∀(*label_i_*, *index_i_*), (*label_j_*, *index_j_*). *label_i_* ≠ *label_j_* ⊕ (*label_i_* = *label_j_* = ? Λ *index_i_* = *index_j_*), where ⊕ stands for logical exclusive disjunction.

That is, vertices either have labels from *Concepts* (no two nodes with the same label) or have question marks as labels and are differentiated by an index.

$E_{s}^{P}$ = {(*from, to, label, characteristic, actionable*) | *from, to* ∈ $V_{s}^{P}$,
  *label* ∈ *Relations* ∪ {?}, *characteristic* ∈ (0,1], *actionable* ≥ 0}

Edges in the pattern have some additional properties, namely *characteristic*—the weight of the edge in the quality of a match (see next subsection)—and *actionable*, indicating whether the agent is able to decide to add the edge in case of a match and in what conditions (see Section 4.4).

### 4.3. Recognizing Situation through Context Matching

By using graph matching algorithms—matching a pattern from the agent's set of patterns against the agent's context graph—an agent is able to detect interesting information or problematic situations and is able to decide on appropriate action to take (Section 4.4).

The pattern $G_{s}^{P}$ *matches* the subgraph $G_{A}^{\prime }=(V^{\prime },E^{\prime })$ (presumably $G_{A}^{\prime }\subseteq CG_{A}$, iff there exists an injective function *f_υ_* : $V_{s}^{P}\to V^{\prime }$, so that the conditions (1) and one of (2a) and (2b) are met simultaneously:

(1) ∀*_υ_^P^ =* (*label, index*) ∈ $V_{s}^{P}$ · *υ^P^* = ? ⊕ *f*(*υ^P^*) = *label*
(2a) ∀( $\upsilon_{i}^{P}$, $\upsilon_{j}^{P}$, *label*,…) ∈ $E_{s}^{P}$. (*f* ( $\upsilon_{i}^{P}$), *f*( $\upsilon_{j}^{P}$), *label* ∈ *E′*, for *label* ∈ *Relations*
(2b) ∀( $\upsilon_{i}^{P}$, $\upsilon_{j}^{P}$, ?) ∈ $E_{s}^{P}$. ∃*label* ∈ *Relations*. (*f*( $\upsilon_{i}^{P}$), *f*( $\upsilon_{j}^{P}$), *label*) ∈ *E′*

That is, every vertex in the pattern matches a different vertex from $G_{A}^{\prime }$ (*f_υ_* is injective), which has the same label for non-generic pattern vertices; and every edge in the pattern matches (has the same label and the adjacent vertices match) an edge from $G_{A}^{\prime }$. Subgraph *G′* should be minimal: it contains no edges or nodes that are not matched by elements in the pattern.

We allow partial matches. A pattern $G_{s}^{P}$ *k-matches* (matches except for *k* edges) a subgraph *G′* of *G*, if conditions (2a) and (2b) above are fulfilled for *m_s_ − k* edges in $E_{s}^{P}$, with *k* ∈ {1*..m_s_ −* 1}, $m_{s}=\left\|E_{s}^{P}\right\|$, and *G′* remains connected and minimal.

The same pattern may match various subgraphs of the context graph. A match *i* between a context pattern $G_{s}^{P}$ and the context graph *CG_A_* of an agent *A* is defined as:

*M_A_*_−_*_si_*( $G_{A}^{\prime }$, $G_{m}^{P}$, $G_{x}^{P}$, *f_υ_*, *k_f_*) $G_{A}^{\prime }$ , $G_{m}^{P}$, $G_{x}^{P}$are graphs: $G_{A}^{\prime }\subseteq CG_{A}$is the subgraph (partially) matched by the pattern, $G_{m}^{P}=(V_{m}^{P},E_{m}^{P})\subseteq G_{s}^{P}$ (matched or solved part) is the part of the pattern that matches $G_{A}^{\prime }$ and $G_{x}^{P}=(V_{x}^{P},E_{x}^{P})$ is the rest of the pattern, which is unmatched. There is no intersection (common nodes or edges) between $G_{m}^{P}$ and $G_{x}^{P}$ (it is therefore possible for $G_{x}^{P}$ to contain edges without containing both of their adjacent vertices). In the case of a partial match, function *f_υ_* is defined as *f_υ_* : $V_{m}^{P}\to V^{\prime }$. The quality of the match is measured by *k_f_*, calculated as:
$k_{f}=\frac{\underset{e_{i}^{P}\in E_{m}^{P}}{\text{∑}}e_{i}^{P}\cdot \text{characteristic}}{\underset{e_{j}^{P}\in E_{s}^{P}}{\text{∑}}e_{j}^{P}\cdot \text{characteristic}}$
  The quality of the match improves with every matched edge and improves more if the matched edge has a high *characteristic* factor.

### 4.4. Deciding on the Appropriate Action

When a partial match occurs between one of the patterns of the agent and the context graph, with a *k_f_* above a certain threshold, it means that the situation corresponding to the pattern has been detected, but it is incomplete. For instance, the person is preparing to leave, but has not taken the keys to the house; or, an emergency situation occurs, but a notification has not yet been sent. Missing edges may lead to creating the edge, in case the system is able/allowed to create the edge, to taking action, in case the system is able to perform an action that results in the creation of the edge; or to notifying the user, in case the system is not able to perform such an action (see Section 6.1 for some examples).

Whether the edge can be inferred/acted upon by the system depends on the *k_f_* value of the match and on the *actionable* property of the edge in question. Namely, if *actionable ≥ k_f_*, then the system is able to add the edge to *CG_A_*. The more complete the match (especially if no characteristic edges are missing), the more able the system to action upon actionable edges. The less actionable an edge (lower *actionable* value), the more complete a match must be in order to lead to the inference of the edge. An edge with zero actionability cannot be acted upon, except if the match is complete, in which case, the edge is already matched.

As an agent may have several actionable edges (across various patterns), choosing which action should be taken at a moment of time, if any, is done using the partial matches of the patterns. When an actionable edge, in a pattern that is otherwise matched, is missing from the context graph, an action will be taken. This is how context patterns can be used to select the action to be performed by the agent.

### 4.5. Time in the Context Graph

Context is dynamic. The user's context changes with the user's activity and other changes in the environment, which are relevant to the user's activity. In order to account for these changes, an agent/a system may probe the environment continuously to get the exact current situation, but this may not be always possible or tractable. However, one agent can assume that some things remain true for a certain amount of time during which they qualify as beliefs (the agent cannot now for sure if they are true).

This mechanism is implemented in the context graph using the *validity* property for edges. For edges that will expire, the property indicates when they will expire. The context graph is associated with a clock, providing a value for the current moment of time (e.g., milliseconds passed since January 1, 1970). Whenever an edge expires, it is removed from the graph.

An edge acquires validity either by being added manually by a user or by being inferred by the system as a result of a match with a *k_f_* larger than the edges' actionability (see Section 4.4). When an edge is inferred, it is added to the graph with a *validity* equal to the *persistence* property of the pattern.

### 4.6. Recognizing Sequences of Events with Timelines

In a context-aware application, and in AAL in particular, there has been a great deal of research in the field of recognizing sequences of activities [45,46,65]. These works show the difficulties in managing temporal sequences. For example, if the system knows just that Emily is in the bathroom, it cannot tell if any problem has occurred. If Emily has been in the bathroom for five minutes, it is alright; if she has been in there for one hour, then it may be likely that there is a problem; conversely, if she goes to the bathroom too often, that should be equally noted.

To this end, we have included in our model a new element—the *timeline*. Timelines integrate with the current framework for graph matching and allow the user to describe sequences of events that should be recognized and the appropriate action to take, if any.

A timeline is a second-order graph pattern (see also Figure 4):

$T_{s}^{P}=(V^{P},E^{P},\text{state})$, with *V^P^* ⊆ *Patterns, state* ∈ {*active, inactive*} and
*E^P^* = {(*from, to, label, state*) | *from*, *to* ∈ $V_{s}^{P}$, *label* ∈ *Labels, state* ∈ {*disabled, enabled, active*}}, with
*Labels* = {(λ), (‘*next*’)}
  ∪{(*relation, quantum*) | *relation* ∈ { ‘*sooner-than*’, ‘*later-than*’}, *quantum* > 0}
  ∪{(‘*interval*’, *quantum*1, *quantum*2) | *quantum*1, *quantum*2 > 0, *quantum*1 < *quantum*2}
  ∪{(‘*time*’, *quantum*) | *quantum* > 0}

A node in a timeline is a pattern in *Patterns*. The label of an edge can take values that are time lengths (e.g., “5 minutes”, “more than 1 hour”, *etc.*), special values (e.g., *next*) or the empty string. Once a timeline is activated, its nodes and edges describe a sequence in which patterns should be matched. It is required that the timeline has one root, which is a single node with no incoming edges.

Initially, a timeline is *inactive* and all edges in it are *disabled*. A timeline becomes *active* when the pattern in the root of the timeline is matched. As a result, the edges going out from the root become *enabled*. Enabled edges can become *active* depending on their label (see below). When the pattern in the node at the destination of an *active* edge is matched, all edges in the timeline become *disabled* and the edges outgoing from the matched node become *enabled*. The process continues, until no edges are *enabled* or *active*, in which case the timeline becomes *inactive*.

Edges that are *enabled* become *active* as follows:

- edges with an empty label are activated immediately after they are enabled and remain so indefinitely (until they are disabled by an active node being matched);
- edges labeled with *next* are activated when the pattern in the source node of the edge is not matched anymore and activate their destination node only if the pattern in the destination node becomes matched at the same time (in the same sequence; for the definition of sequence, see Section 5.1);
- edges labeled with ‘sooner than *time*’ are activated immediately and are disabled after *time*;
- edges labeled with ‘later than *time*’ are activated after *time* passes and remain so indefinitely;
- edges labeled with ‘interval *time*_1_*time*_2_’ are activated after *time*_1_ passes and are disabled after *time*_2_ has passed since the timeline became *active*;
- edges labeled with ‘time *time*’ are activated after *time* has passed and are disabled at the next sequence.

The matching of context patterns works in the same way for timelines as with usual patterns from *Patterns*, with the exception that patterns in the nodes of the timeline are tested if they match only when the node is active. If a matched pattern has actionable edges, the corresponding action will be taken.

## 5. Implementation Details

We have implemented the model presented in the previous section in a piece of software that we called a Continuous Context Matching Platform (CCM platform). It was implemented in Java, starting from the quick graph matching algorithm presented in previous work [8], but with important modifications to deal with persistent matching of a slightly changed context graph against the same set of context patterns. The CCM stays at the core of the context-awareness layer of agents in the multi-agent AmI platform AmIciTy (see Section 3.2).

### 5.1. Efficient Persistent Context Matching

In our previous work, we have developed an algorithm that matches a context pattern against a context graph by first creating single-edge matches (one edge from the pattern to one edge from the graph) and then growing them to achieve maximal matches. The gist of the algorithm is that edge and node labels are compared only when creating the initial matches. Subsequently, a set of correct merge candidates is maintained for each match, so that matches can be merged just by uniting them and aggregating their merge candidate sets.

In the CCM platform, the difference from the previous case is that, in time, the context graph changes only slightly; therefore, a full matching process, against each pattern, is unnecessary. A second aspect deals with sequence. Edges may be added to or removed from the graph in rapid sequence, but it is necessary that the matching is done against a graph that stays constant through the matching process.

**Figure 5.**
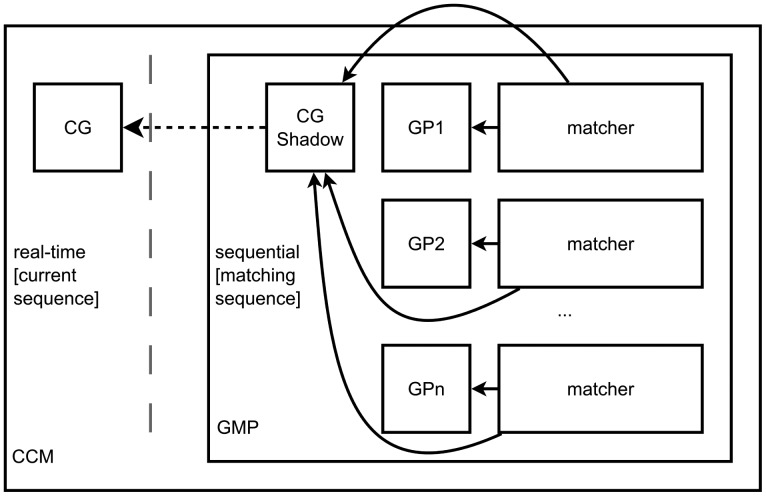
Architecture of the Continuous Context Matching Platform (CCM) for a context graph (*CG*) and various patterns (*GP_i_*), matched using a Graph Matching Platform (GMP).

The CCM platform (see Figure 5) completely deals with the matching of a set of patterns (timelines or not) against a context graph. At any time, the context graph can be modified. Edges in the context graph may have limited or unlimited persistence.

The CCM platform uses, internally, a component that is called a Graph Matching Platform (GMP). The GMP is capable of obtaining all matches of a set of patterns against a context graph. Note that we treat patterns independently, not as a library. The similar, but very different, case would be to match a new graph every time, against a fixed set of patterns [66]. This is done sequentially. Every time the CG changes, the change is recorded as a transaction in a queue of transactions. A transaction can also contain multiple changes to the graph. If all matching is done, the sequence of the GMP is incremented, and the patterns are matched against the context graph with the next transaction applied. In fact, the GMP works on a graph that is a *shadow* of the current CG. In the CCM, the thread registering modifications to the graph is different from the thread in which the GMP runs, so that the GMP has time to perform the matching. Applications can register notification receivers with the CCM, and when a match is found, the receiver is notified, asynchronously. When the match occurs, the CCM also manages any modifications related to actionable items.

Beside the shadow of the CG, the GMP contains the list of patterns, each one having attached to it a Matching Process. Whenever a transaction is applied to the CG shadow, the Matching Processes are notified of each individual change. A Matching Process holds two indexes of matches: for each edge in the graph, the list of matches containing that edge, and for each in the pattern, the list of matches containing it. When an edge in the graph is removed, all matches containing that edge are declared invalid and will be removed whenever they are iterated over. When an edge is added to the graph, initial matches are created (against matching edges from the pattern), and merge candidates are added from among the matches of the neighbor pattern edges. This way, removing an edge is done in *O*(1) (per pattern) and the addition of an edge is done in *O*(*m̅*), where *m̅* is the mean number of neighbor edges for an edge in the pattern, plus the time to grow any new matches (as per the normal graph matching process).

## 6. Scenarios and Experiments

In order to evaluate the context matching platform, we have devised several scenarios and experiments. Context matching was tested both against normal context graphs and against timelines. Let us present in the following sections some of these scenarios, which have proven challenging and interesting to design and implement.

In what follows, we will be using a textual graph representation that we have previously developed [8] for greater facility in representing graphs in text, for a human reader. The representation can easily be translated to ASCII code and can both be produced and interpreted by a computer. In the representation, all edges are represented towards the right. Branches of nodes with multiple children are surrounded by parentheses (except for the last branch). Cycles are marked with a star. The implementation produces a representation, such that the paths are as long as possible. For example, a cycle of three nodes *A, B* and *C* is shown as *A* → *B* → *C* → *A; a tree with a root and two children, with edges labeled 1 and 2, is shown as $A(\overset{1}{\to }B)\overset{2}{\to }C$ (or, in ASCII, A(-1->B)-2->C).

**Figure 6.**
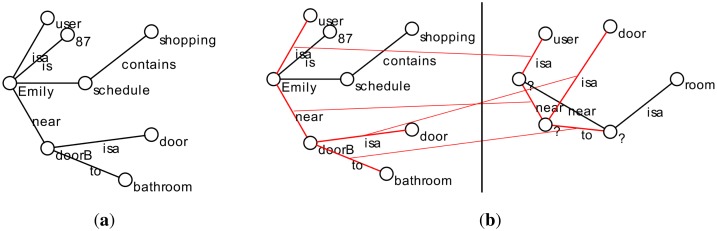
(**a**) Graphical representation of a graph. (**b**) Graphical representation of a match.

The text representation of graphs is at the base of an early-stage user interface, destined to offer the user an easy manner of visualizing, editing and managing graph representations. Based on the linearization of a graph that leads to the text representation, a graphical representation for graphs, patterns and matches has also been developed, as seen in Figure 6. Moreover, by viewing the graph not only graphically, but also in an easy to understand text representation, the user is able to read it easily, copy and paste it between applications, and edit it in text mode.

### 6.1. Studied Scenarios

**Figure 7.**
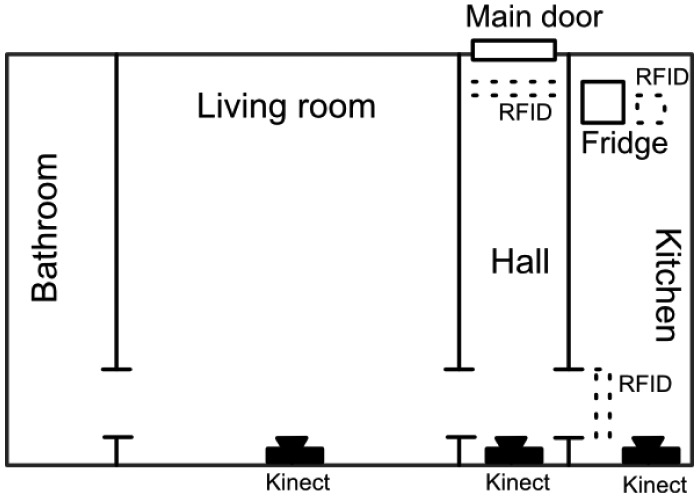
A plan of the house in which the scenarios presented in this paper happen.

All presented scenarios happen in the simulated context of Emily's apartment, a small studio with one room, a hall, the bathroom and the kitchen. In the apartment there are three Kinect^TM^ sensors (room, hall and kitchen) and some RFID readers (apartment door, fridge, kitchen door). A plan is displayed in Figure 7. We present the following scenarios, which have been used to test the platform, in order to give insight into how context graphs can be used in AAL applications.

#### Scenario 1

It is important to keep track of the time Emily spends in the bathroom, especially since there are no sensors inside the bathroom (which makes the scenario more interesting). A notification should be sent to her caretakers if she goes to the bathroom too many times in a day. Furthermore, a potential emergency must be signaled if she stays in the bathroom for too long (longer than one hour).

Both aspects of the scenario can be implemented using the same timeline. The timeline reads:

[*in bathroom*]( $\overset{\leq 5\text{min}}{\to }$ [out of bathroom] $\overset{\text{next}}{\to }$ [log short bathroom visit])
      (→[*out of bathroom*]) $\overset{\geq 1h}{\to }$ [potential emergency]

The timeline is activated once Emily goes into the bathroom. The pattern [*in bathroom*] reads [*Emily* $\overset{\text{near}}{\to }$ *Bathroom*] $\overset{\text{next}}{\to }$ [*system* $\overset{\text{detection}-\text{status}}{\to }$ *not detected*]; that is, Emily is near the bathroom, and next, the system cannot detect her anymore (the representation can be improved by the existence of negative patterns; see Future Work).

If Emily stays in the bathroom for less than five minutes ([*out of bathroom* : *Emily* $\overset{in}{\to }$ *Living Room*]), then the system should log the fact accordingly.

[*log short bathroom visit: System Log* $\overset{\text{entry}/3}{\to }$ *entry*( $\overset{\text{type}/3}{\to }$ *short bathroom visit*) $\overset{\text{date}/3}{\to }$? → *today*]

In the pattern above, actionable edges are marked with /*actionable factor*. The system always contains a node for the current date, linked to the node *today*. If too many different entries of this type exist for the same date, a notification should be created (the representation can be improved by automatically creating multiple instance of the same subgraph; see Future Work):

System(→System Log( $\overset{\text{entry}}{\to }$?^2^( $\overset{\text{type}}{\to }$ short bathroom visit) $\overset{\text{date}}{\to }$?^1^)(…)(…) … $\overset{\text{entry}}{\to }$?^8^( $\overset{\text{type}}{\to }$ short bathroom visit) $\overset{\text{date}}{\to }$?^1^) $\overset{\text{notification}/1}{\to }$ many bathroom visits

The first path in the timeline does not get activated if Emily does not go out of the bathroom after less than five minutes. If she comes out earlier, the second path gets activated, and the timeline ends. However, if more than one hour passes, the system will signal a potential emergency:

[*potential emergency: System* $\overset{\text{notification}/1}{\to }$ *bathroom emergency*]

#### Scenario 2

The platform must help Emily with her shopping list. As smart appliances are expensive and complicated, her caretakers place RFID tags on the products that she buys, and a small RFID reader is placed next to her fridge. Another one is placed next to the trash can. We want the system to assure that there are at least two milk crates in the fridge, to signal if Emily may have forgotten to put the milk away and to add milk to the shopping list if a milk crate is consumed. The timeline is the following:

[2*milk* : *Fridge*( $\overset{\text{contains}}{\to }$ ?^1^ $\overset{is}{\to }$ *Milk*) $\overset{\text{contains}}{\to }$ ?^2^ $\overset{is}{\to }$*Milk] $\overset{\text{next}}{\to }$ [*Fridge* $\overset{\text{contains}}{\to }$?^1^ $\overset{is}{\to }$ *Milk*] →[2*milk*]
[*Trash* $\overset{\text{contains}}{\to }$ ?^2^] →[*Shopping List* $\overset{\text{buy}/1}{\to }$ *Milk*] $\overset{\geq 30\text{min}}{\to }$ [*System* $\overset{\text{notification}/1}{\to }$*Food out of the fridge*]

The *next* edge is activated only when the first pattern is not matched any more; therefore, the timeline will start only when a milk crate will be taken out of the fridge. If it is put back in the fridge, the timeline is reset. If the crate gets into the trash, an item is added to the shopping list. Otherwise, a notification is issued after 30 minutes, warning Emily that she should put the food back in the fridge.

#### 6.2. Experimental Results for 24h-long Scenarios

The Continuous Context Matching Platform presented in Section 5.1 has been tested against various scenarios, among which are the ones in the previous section. Let us present the results of one here, based on the same simulated setup in Figure 7. The 24-hour-long scenario happens over one day in Emily's life. The context graph of the system contains nodes referring to the current state of Emily, the layout of the rooms, food in the fridge, *etc.* (about 50 nodes).

Emily sleeps between 10 pm and 7 am. During the day, she eats two or three times, goes to the bathroom and may take a shower. At times, she wanders around the house and spends time in the kitchen without eating anything, just sitting down and looking out of the window.

##### Generating scenarios

**Figure 8.**
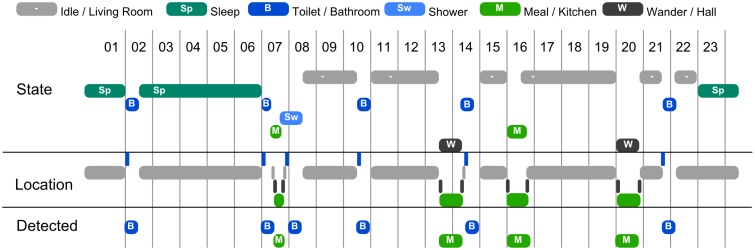
Actual and detected states in Emily's activity, in the experiments presented in Section 6.2. States are horizontally organized by the time (hour) at which they occur and vertically organized by their type. The top set of states (“State”) are the actual simulated activities; the middle set of states (“Location”) are the detected locations of Emily (edges *Emily* $\overset{is-in}{\to }$?); the bottom set of states are the activities detected by the systems. The activities are arranged vertically in the order *Idle-Sleep-Bathroom-Shower-Meal-Wander*; locations are *Near_bathroom-Living_room-Hall-Kitchen*; detected activities are *Bathroom* and *Meal*.

In order to generate a 24-hour-long scenario, we use a generator that inserts various activities at various moments of time, using a distribution of probability for each type of activity. For example, if Emily just ate, she will not eat again for the following three or four hours, but after that, the probability of her deciding to eat will increase with time. For this presentation, we have created scenarios containing six types of activities: sleeping, having a meal, going to the bathroom, taking a shower, wandering around the house and doing nothing. Depending on their type, some activities cannot be interrupted (such as a shower), some can (such as going to the bathroom in the middle of a meal) and some do not need to be continued if interrupted (such as wandering around). Depending on their type, some activities may have variable durations. A visualization of such a generated scenario is presented in Figure 8.

The purpose of the presented experiment is to evaluate how the system recognizes Emily's activity based solely on primitive location detection (e.g., motion sensors). The execution of the scenarios provides location information regarding the room in which Emily is situated (depending on her activity). There are no sensors in the bathroom, but there is an indication of when she is near the bathroom door.

##### Results

The system recognizes several of the various patterns related to Emily's activities. We focus this presentation on the patterns related to going to the bathroom and taking a meal (see also Section 6.1). The detected activity can be seen in the lower part of the visualization in Figure 8.

Out of the two basic situations that are detected, both can be confused with other activities. Taking a shower can be confused with just going to the toilet, but this can be easily fixed by specifying in the timeline for going to the toilet that the activity has a maximum duration. However, a longer stay in the bathroom is a potential problem regardless of the activity Emily was supposed to perform in there. As for meals, the system cannot differentiate between taking a meal and Emily just going to sit in the kitchen and to look out the window. This can be addressed by more complex patterns, either by analyzing the usage of food items in the kitchen or by perceiving the degree of movement of Emily in the kitchen.

We consider that the results obtained, even in such relatively simple scenarios, are promising, showing the flexibility of graph patterns in what regards situation detection, including situations that are defined over a duration of time.

##### System performance

**Figure 9.**
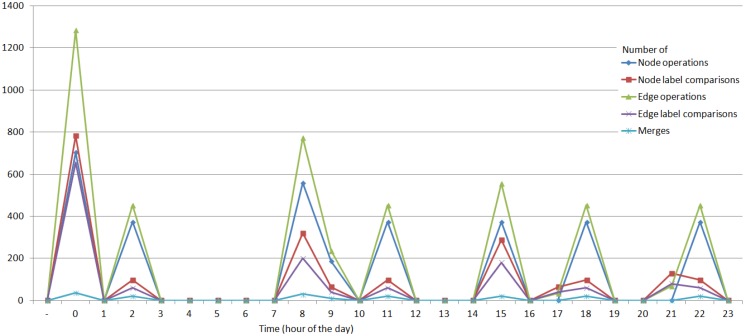
A plot of the number of compared node references and labels, edge references and labels and match merges, depending on the time in the simulated scenario in Section 6.2.

Figure 9 shows some performance indications for the system's activity. The performance indication has been broken down into five tracks: the number of compared node/edge references, the number of compared node/edge labels and the number of merges between existing matches.

One can notice that the largest load on the system is at the start of the system, when the initial matches are created. The load on the system increases also when Emily engages in various activities (e.g., taking a meal). In all cases, note that the number of label comparisons (which is more computationally expensive than reference comparisons and set operations) is significantly lower than the other operations.

Overall, the tasks done by the system are not very computationally intensive. Moreover, in a multi-agent setup, where tasks are divided among agents, the total number of operations is even lower than in the centralized case.

### 7. Conclusion and Future Work

In the field of ambient assisted living, as in ambient intelligence, context awareness is vital in situation detection and reasoning. Context is not only instantaneous, but also temporal, depending on situations that have been detected before.

This work advocates the use of context graphs, context patterns and continuous, persistent context matching as efficient mechanisms for situation detection in AAL, also allowing for a user-friendly interface directly to the core of the system and a reasoning process that is easy to understand.

Context graphs have a solid foundation in graph theory, and graph matching has been researched for a long time. The algorithms and CCM platform that we have developed make context graphs an efficient solution that balances computational effort with memory usage and has a reduced footprint that depends directly on the complexity of the graph and, therefore, on the function of the agent.

The current limitations of the context graph and pattern formalism are related to its structural simplicity, on the one hand, and to temporal relations, on the other. The tradeoff of simplicity is that context patterns cannot account for a model of the environment; therefore, no true anticipation is yet possible. Temporal relations need to be advanced to account for absolute time intervals and coordinates, the frequency of actions, and so on.

Future work for the medium term involves improving the representation, so as to allow working with negative matches (“there is no match for the pattern”) and to make it easier to deal with numbers. Simultaneously, the platform will be tested in the Ambient Intelligence Laboratory, using both single-and multi-user scenarios.

An interesting subject related to context patterns is that of activity detection, learning and the automated discovery of context patterns. Here, previous work by the authors and other research groups can draw from the fields of data mining and behavioral patterns in order to grow patterns as they are recognized. Knowledge may also be discovered by using measures of graph entropy [67] applied to both existing patterns and the current context graph.
